# Pattern and prevelence of alloimmunization in multiply transfused patients with sickle cell disease in Nigeria

**DOI:** 10.1186/s40364-015-0050-3

**Published:** 2015-10-13

**Authors:** Umar Kangiwa, Obike Ibegbulam, Sunday Ocheni, Anazoeze Madu, Ndakosu Mohammed

**Affiliations:** Department of Haematology, University of Nigeria Teaching Hospital, Enugu, Nigeria

**Keywords:** Sickle cell disease, Multiple transfusion, Allo-immunization, Autoantibodies

## Abstract

**Background and study objectives:**

Blood transfusion is central in the prevention and treatment of certain chronic complications of sickle cell disease. It is indispensible in correcting anaemias as well as in the practice of exchange blood transfusion. These gains are largely limited by formation of allo-antibodies. Several studies demonstrated varying frequencies of allo-immunization in various patient groups. The effect of the racial differences between the donor and recipient pool, which has been subsumed in this study, has continuously created a confounding effect on the results of previous studies.

**Aim:**

This study was aimed at determining the pattern and frequency of allo-immunization in multiply transfused sickle cell patients, in a racially matched donor and recipient population.

**Patients and methods:**

This was a cross-sectional case-controlled study involving 80 Nigerian sickle cell disease patients who had received three or more units of packed red cells in the within 4 weeks of the study and 40 controls (who were SCD that had not been transfused in their life time). Antibody screening and identification was done using the Diamed microtyping system.

**Results:**

Frequency of allo-immunization was determined to be 18.7 % (15/80) among the previously transfused and 5 % (15/120) in all sickle cell disease patients. Auto-antibodies were detected in 1.25 % of the study group and 2.5 % of the control, and all reacted with the Kell and Lutheran blood group antigens. The pattern of allo-antibodies found showed; 46.7 % Rhesus, 40 % Kell, while Lutheran and Duffy 13.3 %, each.

**Conclusion:**

Sickle cell disease patients are particularly susceptible to development of allo-antibodies despite racial similarities between the donor and recipient population. The most common allo-antibodies are Rhesus, Kell and Lutheran and Duffy respectively in order of decreasing frequency. Development of auto-antibodies seems to be independent of blood transfusion in sickle cell disease with possibly different pathogenetic mechanism. Policy on extended red cell phenotyping for common antigens will reduce allo-immunization among multiply transfused patients.

## Background

Sickle cell disease (SCD) is an inherited qualitative abnormality of haemoglobin and the most frequently occurring genetic disease afflicting mankind [1–5]. This condition has the highest prevalence in the Equatorial region of Africa, though the gene has a widespread distribution, affecting people of European, Arabian, Indian and Oriental ancestry [1, 3]. About 300,000 children are born annually with SCD woridwide [1], of which 100,000 are born to Nigerian parents. Millions of people are affected worldwide, making it a major public health problem on a global scale.

Sickling of red blood cells, vaso-occlusion and susceptibility to infections are the hallmark of the pathological processes in SCD. Risk factors for sickling include hypoxia, acidosis and rise in temperature, stress and hyper-osmolality. Background haemolysis of the sickle red cells lead to marrow hyperplasia with consequent anaemia. This is further worsened by any superimposed stress on erythropoiesis; like malaria, bacterial infections and acute sequestration, thus necessitating transfusion.

Medical management includes a wide range of treatment modalities ranging from vaccination against capsulated organisms, use of antibiotics to treat infections, analgesics to treat painful episodes, fluid administration to correct and prevent dehydration, regular folic acid supplementation, prophylactic anti-malarial and blood transfusion among others [1]. Transfusion of red blood cells (RBCs) is a common practice in the treatment of patients with sickle cell disease (SCD) such that most adult patients with SCD have been transfused at one time or another, many with large number of units of blood.

Red cell transfusion is usually not required in steady state patients but blood transfusion remains the mainstay therapy for many of the complications of SCD [6–8], however it is associated with complications such as iron overload, transfusion transmitted infections, acute lung injury, anaphylaxis, and allo-immunization [7–9]. The frequency of development of allo-antibodies in transfused patients with sickle cell disease has been studied in many parts of the world and the risk of development of red cell allo-immunization also has been found to be high with overall incidence ranging from 18 to 36 % [1, 10–13].

Sixty six percent of the allo-antibodies identified in most of the reports were those of the Rhesus (Rh) and Kell systems in SCD [1, 11, 14–16]. Some studies identified allo-antibodies of the Kidd and Duffy systems in addition to the Rh and Kell systems. Several studies have also confirmed that matching for major Rh and Kell antigens prevent formation of allo-antibodies in 53.3 % of patients and further extended typing to include MNS, Duffy (Fy^a^) and Kidd blood groups will prevent allo-immunization in 70.8 % of patients [1, 14, 17, 18]. This leaves at least one-third of the allo-antibodies formation still unaccounted for. These previous studies were also done without taking cognizance of the racial disparity between donors and recipients, a factor which definitely impacts on the propensity to form allo-antibodies.

The purpose of this study therefore, is to characterize the allo-antibodies formed with racially matched blood given to multiply transfused (more than two units) patients with sickle cell disease. This would effectively check the confounding effect of the racial differences in distribution of red cell antigens. It also aims to ascertain the prevalence of allo-immunization and evaluate the specificity as well as the clinical relevance of the allo-antibodies found.

## Patients and methods

This was a prospective cross-sectional study carried out at the adult and paediatric sickle cell clinics of the University of Nigeria Teaching Hospital Ituku Ozalla, Enugu Nigeria.

### Patients recruitment

One hundred and twenty patients from both adults and paediatric sickle cell clinic who gave written informed consent were recruited for the study. In the case of paediatric patients, informed consent was obtained from their parents or guardian. A standard questionnaire was completed by the patient (or their care givers, in the case of minors) during the recruitment process before entry into the study. They were divided into two groups according transfusion history obtained from a questionnaire.

### Data collection

Group 1 (Study Group) subjects were 80 previously diagnosed SCD patients who had received more than two units of red cells transfusion in their life time and the last unit of blood received was more than 4 weeks prior to sample collection. The sample size of 80 for the study group was obtained using GraphPad Statmate 2.0, prevalence rate of sickle cell anaemia in Nigeria being noted to be 0.026 in previous studies.

Group 2 (Control Group) subjects were 40 SCD patients who had never been transfused as determined by history and review of case notes.

Antibody screening was carried with Diamed Microtyping System, using a 6-well gel card each containing low ionic saline solution (LISS/ coombs card). 50 μl of the commercially prepared Diacels I, II, and III, was added into corresponding wells for the test, 25 μL of the test plasma was added into each of the 6 wells. 50 μL of the patients cell suspension was added into the commercially prepared cells from Diamed well of auto control.

Diacells I and II were added into the last two wells containing enzymes. The gel card was incubated for 15 min using Diamed incubator, at 37 °C and then centrifuged for 10 min at 1000p/min using Diamed centrifuge. The results were read both visually and by the use of computerized ID – reader of the Diamed- cooperation. The cells that are agglutinated (positive reaction) remained trapped in the gel while those that are not agglutinated (negative reaction) passed through the beads of the gel to the bottom of the well.

Ethical approval was obtained from the University of Nigeria Teaching Hospital Health Research and Ethics review committee.

### Statistics

The relationship between the age of the patients and the presence of antibodies (allo, auto or both was calculated using the Pearsons and Kendaull tau_b correlation coefficient. Significance was set at all values less 0.05. While the relationship between the sex of the patients and the presence of antibodies ws calculated using the Chi Square and Fischers exact test. The Statistical Package for Social Sciences (SPSS) version 17.0 (Chicago IL) was used for data analysis and the results obtained were expressed in figures and tables.

## Results

**Fig. 1:**
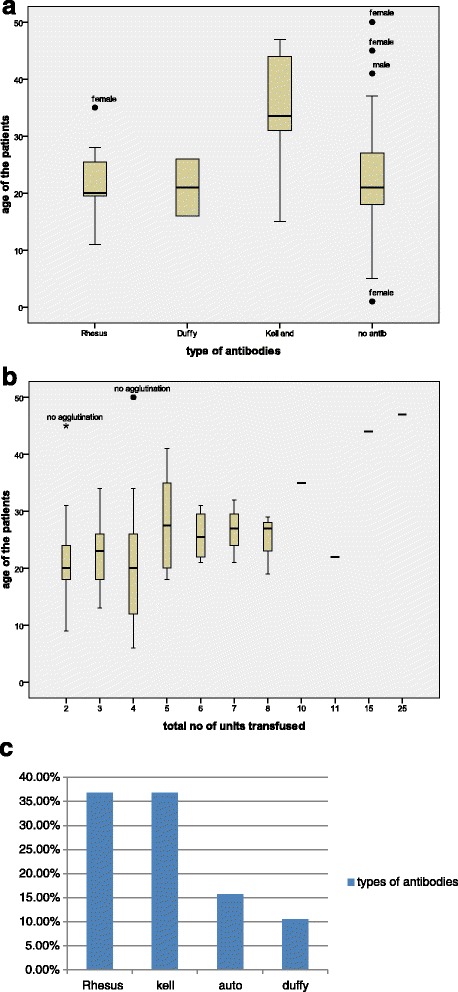
**a** Distribution of age and types of antibodies detected in the transfused sickle cell patients. **b** Distribution of the total number of units of blood and ages of patients amongst sickle cell anaemia patients. **c** Frequency of occurrence of the various types of antibodies observed in sickle cell patients

Antibody screening was carried out on 120 SCD patients, divided into the study and control group. The patients were aged 1–50 years with a median age of 21 years. Eighty of the patients had been transfused in the past while 40 of them had not been previously transfused. In all, there were 59 males and 61 females, while among the 80 previously transfused patients, there were 35 males and 45 females. Of those transfused, 44.2 % had their last transfusion more than a month ago and their median age as at the first transfusion was 8 years. The age and sex distribution of the patients in this study are shown on Fig. 1a.

The prevalence of allo-immunization among sickle cell patients was determined to be 5 % (15/120). There seemed to be some relationship, though not statistically significant, between increasing age and presence of antibodies (*p* = 0.068). There was also no relationship observed between the sex of the patients and the development of antibodies, Chi square value 0.033 (*p* = 0.855).

While the prevalence of allo-immunization amongst the patients who had been previously transfused with racially matched blood was 18.7 % (15/80). The transfused patients had received an average of 3 units of red cells in the past. Nineteen (23.8 %) of the previously transfused patients had experienced various forms of transfusion reaction. There had also been difficulty in cross-matching blood for 3 (3.8 %) of them, and there was no association between the age or sex of patients in this group and the occurrence of antibodies (*p* =0.519 and 0.264, respectively). The age and sex distribution of the patients as well as the number of units of red cell s transfused are shown in Fig. 1b.

Thirty of the patients, all from the study group had anti-red cell antibodies (either auto-, allo- or both forms of red cell antibodies). One patient had a solitary positive Coombs test, 15 patients had combined positive Coombs (auto-antibodies) and red cell antigen tests (allo-antibodies), 11 patients had positivity only for the red cell antigen test, while 90 patients had double negative (absence of both auto and allo-antibodies) result. Of the 30 patients that reacted with antibodies during screening, only 15 allo-antibodies and 3 auto antibodies were found. No allo-antibody was identified from the 11 patients that reacted positively with enzymes of gel cards alone, these were false positives. There is increased frequency of false positive results in the use of enzymes alone in antibody screening, Table 1.

**Table 1:** Frequency and pattern of agglutination in sickle cell patients

Antibody screening				
	Frequency	Percent	Valid %	Cumulative %
Agglutination with Coombs reagent only	1	0.8	0.8	0.8
Agglutination with Coombs reagent + enzymes	15	12.5	12.5	13.3
Agglutination with enzyme only	11	9.2	9.2	22.5
Agglutination with auto cells	3	2.5	2.5	25.0
No agglutination	90	75.0	75.0	100.0
Total	120	100.0	100.0	

None of the patient in the control group had developed red cell allo-antibodies. Blood transfusion is significantly associated with allo-immunization, *P* = 0.000. Of the 3 auto-antibodies identified, one was from study group and the other two were from control group. Antibody Identification of the 15 allo-antibodies found revealed that, 7 (46.7 %) patients reacted positively to Rhesus blood group system, 1 (6.7 %) specifically to D antigen, 2 (13.3 %) to E antigen, 2 (13.3 %) to c antigen and 2 (13.3 %) to e antigen. 2 (13.3 %) of the patients reacted positively to Duffy blood group system, 1(6.7 %) specifically to Fy^a^ antigens and 1 (6.7 %) to Fy^b^ antigens. 6 (40 %) patients reacted positively to the combination of k, kp^b^ and Js^b^ antigens of the Kell blood group as well as Lutheran (Lu^a^) antigens of the Lutheran blood group, Fig. 1c. All the auto antibodies reacted to Kell as well as Lutheran antigens, Table 2.

**Table 2:** Frequency and pattern of allo-antibodies observed

Antibody reactions and types of antibody						
	Types of antibody					
Antibody screening	Rh	Duffy	Kell	No. of antibody	Auto antibody	Total
Agglutination with Coombs reagent only	0	0	1	0	0	1
Agglutination with Coombs reagent + enzymes	7	2	5	1	0	15
Agglutination with enzyme only	0	0	0	9	0	9
Agglutination with auto cells	0	0	0	0	1	1
No agglutination	0	0	0	54	0	54
Total	7	2	6	64	1	80

Allo-antibodies were also found in all the patients who developed auto-antibodies. Sixty one females were involved in the study of which only three were married and all the three had alloantibodies.

## Discussion

Development of allo-immunization depends on number factors such as racial difference between the donor population and the recipient, number of blood units transfused, age of the patient, severity of the disease and Hb genotype among other things. This study provides information on the prevalence of allo-immunization in a racially identical donor-recipient population, thus ameliorating for the effects of antibodies formed as a result of dono-recipient racial discrepancy.

In this study 18.7 % developed allo-immunization among multiply transfused patients even though both the donor and the recipients have similar racial background and none among non-transfuse patients. The prevalence of allo-immunization in our study is slightly higher to prevalence in other studies done in Nigeria 8.8 % in Kano and 3.2 % in Lagos. This may be attributed to the sensitivity (automated gel technology) of the method used in this study as opposed to manual visual method used in other studies. The use of Coombs reagents with enzymes - papain and bromelin, which were used in this study enhances reactivity of some antibodies such as Rh, P, I, Kidd and Lewis, explaining the higher detection rate of the antibodies as compared with other studies. The results obtained using these reagents were; 12.5 %, using Coombs reagent with papain and bromelin, compared to Coombs reagents only 0.8 % and enzymes only 9.2 %, which implies a variation in reactivity.

Several international studies showed a wide variation in frequencies of allo-immunization in sickle cell disease such as 6.1 % in Uganda, 29 % in US, 21 % in UK, 30 % in Oakland US, 12.0 and 41 % respectively in two separate reports from Brazil [19–22]. In general, the frequency ranges between 18 and 36 % in most of the studies [23]. Our data concur with the results of many studies that allo-immunization occurs in multiply transfused patient with sickle cell disease with the same racial background between the donor and recipient population. These findings further reaffirm previous observations that multiple blood transfusion is associated with development of alloimmunisation even in racially matched donors and recipient.

The development of auto-antibodies seems to be independent of allo-immunization or blood transfusion. In this study auto-antibodies were detected in three persons;, one in multiply transfused patients and two in non transfused group. This further conforms to the findings of other studies. Several studies have shown that D, C/c, E/e, and K antigen matching decreases the risk of alloimmunization [24, 25], Even with such phenotype antigen matching, alloimmunization is still a major problem. This suggests that the development of autoantibodies in sickle cell disease may be due to genetic respondents with an increased tendency to develop erythrocyte antibodies or due to exposure of neo-antigens from repeated destruction of erythrocyte membrane that induces IgG autoantibody formation.

## Conclusion

Multiply transfused patient with sickle cell disease are prone to development of allo-antibodies despite similar racial background between the donor and recipient population. The prevalence of allo-immunization amongst multiply transfused sickle cell patients who received racially matched blood was found to be 18.7 %. Lack of extended red cell typing among multiply transfused patients further predisposes to allo-immunization. The development of auto antibodies appears to be independent of blood transfusion in sickle cell disease patients. The age of the patient and multiple blood transfusions are significantly associated with the development of allo-immunization, *P* = 0.000.

## References

[1] Okpala I (2004). Epidemiology, genetics and pathophysiology of sickle cell disease. Practical management of haemoglobinpathies.

[2] Sergeant GR (1997). Sickle cell disease. Lancet.

[3] Wheatherall DJ, Clegg JB (2001). Inherited haemoglobin disorders: an increasing global health problem. Bull World Health Organ.

[4] Davies SC, Brozovic M (1989). The presentation, management and prophylaxis of sickle cell disease. Blood Rev.

[5] Escoffery CT, Suzanne ES (1998). Autopsy findings and causes of death in sickle cell disease. Postgraduate Doctor Africa.

[6] Wanko SO, Telen MJ (2005). Transfusion management in sickle cell disease. Hematol Oncol Clin North Am.

[7] Vichinsky EP, Haberkern CM, Neumayr L, Earles AN, Black D, Koshy M (1995). A comparison of conservative and aggressive transfusion regimens in the perioperative management of sickle cell disease. The Preoperative Transfusion in Sickle Cell Disease Study Group. N Engl J Med.

[8] Danielson CF (2002). The role of red blood cell exchange transfusion in the treatment and prevention of complications of sickle cell disease. Ther Apher.

[9] Neumayr L, Koshy M, Haberkern C, Earles AN, Bellevue R, Hassell K (1998). Surgery in patients with hemoglobin SC disease. Pre-operative Transfusion in Sickle Cell Disease Study Group. Am J Hematol.

[10] Vichinsky EP, Luban NL, Wright E, Olivieri N, Driscoll C, Pegelow CH (2001). Prospective RBC phenotype matching in a stroke- prevention trial in sickle cell anemia: a multicenter transfusion trial. Transfusion (Paris).

[11] Murao M, Viana MB (2005). Risk factors for alloimmunisation by patients with sickle cell disease. Baz J Med Bio Res.

[12] Sarnaik S, Schornoack J, Lusher JM (1986). The incidence of development of irregular red cell antibodies in patients with sickle cell anaemia. Transfusion.

[13] Ygun BA, Padmanabhan S, Paley C, Visalam C (2002). Clinical significance of RBC alloantibodies and autoantibodies in sickle cell patients who received transfusion. Transfusion.

[14] Rosse WF, Gallagher D, Kinney TR, Castro O, Dosik H, Moohr J (1990). Transfusion and alloimmunisation in Sickle Cell Disease, the cooperative study of Sickle Cell Disease. Blood.

[15] Norol F, Nadjahi J, Bachir D, Desaint C, Guillou Bataille M, Beaujean F (1994). Transfusion and alloimmunisation in sickle cell anaemia patients. Transfusion Clin Biol.

[16] Castro O (1999). Management of sickle cell disease: recent advances and controversies. Br J Haematol.

[17] Castro O, Sandler SG, Houston-Yup RS (2002). Predicting the effect of transfusion only phenotype matched RBCS to patients with sickle cell disease: theoretical and practical implications. Transfusion.

[18] Vichinsky EP, Earles A, Johnson RA, Hoag MS, Williams A, Lubin B (1990). Alloimmunisation in sickle cell anaemia and transfusion of racially unmatched blood. N Engl J Med.

[19] Konotey-Ahulu FID (1991). Sickle cell disease patients Macmillan Books, Lagos.

[20] Konotey-Ahulu FID (1968). Hereditary qualitative and quantitative erythrocytes defects in Ghana: an historical and geographical survey. Ghana Med J.

[21] Searjent GR (2001). Sickle cell disease. Third edition.

[22] Pauline L, Itano HA, Singer SJ, Wells IC (1949). Sickle cell anaemia, a molecular disease. Science.

[23] Graham RS, Beryl ES (2001). The epidemiology of sickle cell disorder: a challenge for Africa. Achieves of Ibadan Medicine.

[24] Miller ST, Kim HY, Weiner DL, Wager CG, Gallagher D, Styles LA (2013). Investigators of the Sickle Cell Disease Clinical Research Network (SCDCRN). Red blood cell alloimmunization in sickle cell disease: prevalence in 2010. Transfusion.

[25] Chou ST, Jackson T, Vege S, Smith-Whitley K, Friedman DF, Westhoff CM (2013). High prevalence of red blood cell alloimmunization in sickle cell disease despite transfusion from Rh-matched minority donors. Blood.

